# Oxidative Stress as A Mechanism for Functional Alterations in Cardiac Hypertrophy and Heart Failure

**DOI:** 10.3390/antiox10060931

**Published:** 2021-06-08

**Authors:** Anureet K. Shah, Sukhwinder K. Bhullar, Vijayan Elimban, Naranjan S. Dhalla

**Affiliations:** 1 School of Kinesiology, Nutrition and Food Science, California State University, Los Angeles, CA 90032, USA; akaur23@calstatela.edu; 2 Institute of Cardiovascular Sciences, St. Boniface Hospital Albrechtsen Research Centre, Department of Physiology and Pathophysiology, Max Rady College of Medicine, University of Manitoba, Winnipeg, MB R2H 2A6, Canada; SBhullar@sbrc.ca (S.K.B.); VElimban@sbrc.ca (V.E.)

**Keywords:** vasoactive hormones, cardiac hypertrophy and failure, myocardial infarction, metabolic derangements, myocardial inflammation, oxidative stress, Ca^2+^-handling abnormalities

## Abstract

Although heart failure due to a wide variety of pathological stimuli including myocardial infarction, pressure overload and volume overload is associated with cardiac hypertrophy, the exact reasons for the transition of cardiac hypertrophy to heart failure are not well defined. Since circulating levels of several vasoactive hormones including catecholamines, angiotensin II, and endothelins are elevated under pathological conditions, it has been suggested that these vasoactive hormones may be involved in the development of both cardiac hypertrophy and heart failure. At initial stages of pathological stimuli, these hormones induce an increase in ventricular wall tension by acting through their respective receptor-mediated signal transduction systems and result in the development of cardiac hypertrophy. Some oxyradicals formed at initial stages are also involved in the redox-dependent activation of the hypertrophic process but these are rapidly removed by increased content of antioxidants in hypertrophied heart. In fact, cardiac hypertrophy is considered to be an adaptive process as it exhibits either normal or augmented cardiac function for maintaining cardiovascular homeostasis. However, exposure of a hypertrophied heart to elevated levels of circulating hormones due to pathological stimuli over a prolonged period results in cardiac dysfunction and development of heart failure involving a complex set of mechanisms. It has been demonstrated that different cardiovascular abnormalities such as functional hypoxia, metabolic derangements, uncoupling of mitochondrial electron transport, and inflammation produce oxidative stress in the hypertrophied failing hearts. In addition, oxidation of catecholamines by monoamine oxidase as well as NADPH oxidase activation by angiotensin II and endothelin promote the generation of oxidative stress during the prolonged period by these pathological stimuli. It is noteworthy that oxidative stress is known to activate metallomatrix proteases and degrade the extracellular matrix proteins for the induction of cardiac remodeling and heart dysfunction. Furthermore, oxidative stress has been shown to induce subcellular remodeling and Ca^2+^-handling abnormalities as well as loss of cardiomyocytes due to the development of apoptosis, necrosis, and fibrosis. These observations support the view that a low amount of oxyradical formation for a brief period may activate redox-sensitive mechanisms, which are associated with the development of cardiac hypertrophy. On the other hand, high levels of oxyradicals over a prolonged period may induce oxidative stress and cause Ca^2+^-handling defects as well as protease activation and thus play a critical role in the development of adverse cardiac remodeling and cardiac dysfunction as well as progression of heart failure.

## 1. Introduction

**Figure 1:**
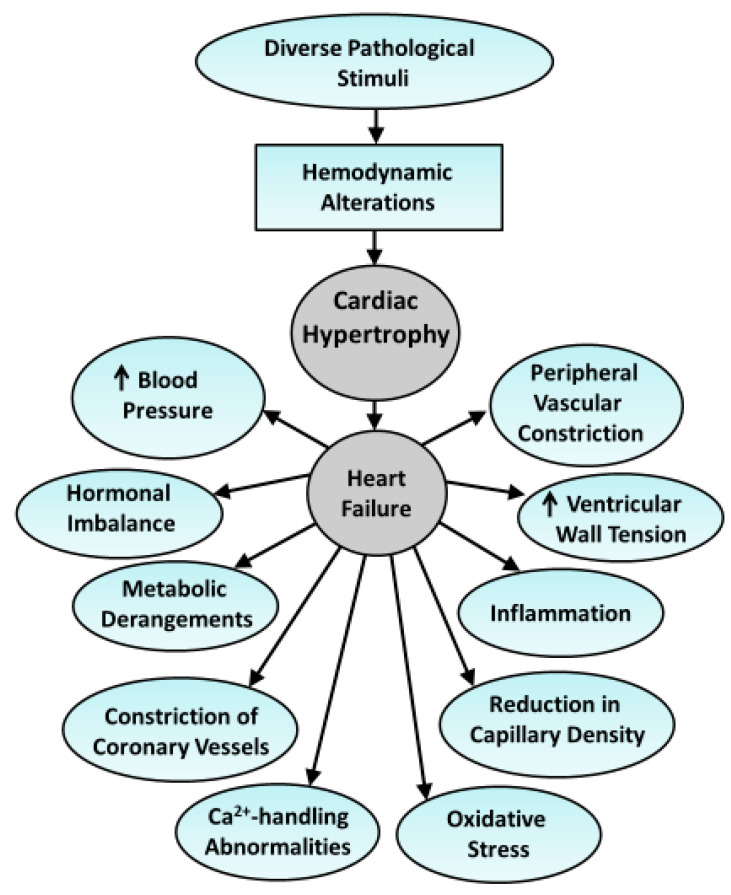
Schematic representation for the development of cardiac hypertrophy and heart failure as well as associated cardiovascular abnormalities due to diverse pathological stimuli.

Heart failure due to several pathological conditions such as myocardial infarction, hypertension, valvular defects, diabetes, atherosclerosis, and different types of cardiomyopathies, is invariably associated with cardiac hypertrophy [1,2,3,4,5,6]. Extensive research regarding hemodynamic, cellular, and biochemical mechanisms have revealed that heart failure may be due to loss of cardiomyocytes, adverse cardiac remodeling, defects in subcellular activities, Ca^2+^-handling abnormalities, alterations in myocardial metabolism and elevation of different hormones in the circulation [7,8,9,10,11,12]. Some of these cardiovascular alterations and mechanisms associated with the development of cardiac hypertrophy and subsequent heart failure are shown in Figure 1. It is becoming clear that all these mechanisms for the induction of contractile defects are inter-related and cardiac dysfunction is the hallmark for identifying the development of heart failure [13,14,15]. On the other hand, cardiac function of hypertrophied heart is either normal or increased at early stages whereas cardiac performance is impaired in heart failure [16,17,18,19]. Although excellent review articles regarding the molecular and cellular mechanism for the development of cardiac hypertrophy and heart failure are available in the literature [20,21,22,23,24], exact reasons for the occurrence of cardiac dysfunction in hypertrophied heart are not fully understood [25,26]. It is therefore important to gain some information to understand the mechanisms involved in the transition of cardiac hypertrophy to heart failure as well as pathophysiology of cardiac dysfunction during the progression of heart failure.

Although elevated levels of several vasoactive hormones in the circulation due to activation of sympathetic nervous system, renin–angiotensin system, and other neuro-endocrine systems have been shown to occur in different types of heart failure [27,28,29,30], the mechanisms for their beneficial actions for the development of cardiac hypertrophy and adverse effects for the occurrence heart failure are not well understood. It is generally held that the acute effects of elevated vasoactive hormones increase cardiac muscle mass, add contractile units, and produce cardiac hypertrophy upon stimulating their respective receptor-mediated signal transduction pathways. Furthermore, these acute effects are associated with the formation of low amounts of oxyradicals and activation of redox-sensitive signal transduction for the occurrence of cardiac hypertrophy [7,12,15,16]. Myocardial hypertrophy in response to diverse stimuli is an adaptive process where cardiac performance and subcellular organ function are either normal or augmented to maintain hemodynamic homeostasis indicating the beneficial effects of vasoactive hormones [13,16,18,26]. On the other hand, prolonged exposure of hypertrophied heart to elevated levels of vasoactive hormones due to pathological stimuli for a prolonged period has been shown to induce metabolic derangements, Ca^2+^-handling abnormalities, protease activation, subcellular defects, and cardiac dysfunction leading to the development of heart failure [11,12,13,14,15,17,26]. Thus, it appears that cardiac hypertrophy and heart failure represent two different stages of effects, namely adaptive cardiac remodeling and adverse (maladaptive) cardiac remodeling, initiated by diverse pathological stimuli. The present article is therefore intended to describe some salient features for the development of both cardiac hypertrophy and heart failure as a consequence of some pathological situations including myocardial infarction, pressure overload and volume overload. It is also planned to discuss different mechanisms involved in the generation of oxyradicals for the activation of redox-sensitive hypertrophic process as well as the development of oxidative stress. The consequence of oxidative stress for the transition of cardiac hypertrophy to heart failure and pathophysiology of hypertrophied heart during progression of heart failure due to adverse effects of elevated levels of some vasoactive hormones for a prolonged period will be highlighted. In addition, efforts will be made to describe some of the subcellular and metabolic abnormalities under some experimental conditions, which are known to promote the occurrence of oxidative stress and induce cardiac dysfunction in non-hypertrophied hearts. The effectiveness of different oxyradical scavengers and antioxidants on the experimentally induced cardiac dysfunction as well as subcellular defects will be examined. Evidence will also be presented to show both direct and indirect effects of oxidative stress on subcellular organelles and Ca^2+^-handling abnormalities associated with heart dysfunction. 

## 2. Development of Cardiac Hypertrophy and Heart Failure

Over the past 30 years various types of pathological stimuli have been shown to involve different signal transduction pathways as well as cellular and molecular mechanisms for the genesis of cardiac hypertrophy and heart failure [5,6,7,8,9,10,20,21,22,23,24,25,26]. Despite difference in the patterns of signal transduction mechanisms, there are several similarities in cardiovascular alterations, which occur during the initial and later stages of cardiac hypertrophy as well as heart failure. Various vasoactive hormones and growth factors are elevated not only for stimulating cardiovascular function and maintaining blood supply to all organs of the body but also for the induction of cardiac hypertrophy [27,28,29,30,31,32,33]. Although cardiac hypertrophy as a consequence of increased muscle mass has been shown to be of hypertrophic type or dilated type depending upon the pathological stimulus, both forms of cardiac growth have been reported to be either physiological or pathological in nature depending upon the type as well as duration and magnitude of the stimulus [13,14,15,16,17,25,26]. It appears that physiological hypertrophy is concerned with improving cardiac performance due to increased number of contractile units and augmented function of subcellular organelles whereas pathological hypertrophy associated with cardiac dysfunction may represent a pre-failure stage or reflect the transition of hypertrophied myocardium to heart failure. It should also be mentioned that there occurs a progressive increase in the levels of some vasodilatory natriuretic peptides (ANP and BNP) as well as endothelial nitric oxide (NO) in the circulation to maintain hemodynamic homeostasis; in fact, both ANP and BNP are commonly used as biomarkers for characterization of the heart failure stage [34,35,36]. On the other hand, prolonged exposure of hypertrophied heart to elevated levels of vasoactive hormones is considered to result in the progression of adverse cardiac remodeling and heart failure. Thus, it appears that cardiac hypertrophy and heart failure due to diverse pathological situations are associated with acute and chronic effects of the elevated levels of vasoactive hormones, respectively.

### 2.1. Development of Heart Failure Due to Myocardial Infarction

Myocardial infarction is known to produce loss of a portion of the ventricular tissue due to ischemia and is known to be the major cause of heart failure. The development of both cardiac hypertrophy and heart failure due to myocardial infarction are dependent upon the duration and size of infarct in the heart. The hemodynamic alterations such as decreased blood pressure and reduced cardiac output at initial stages activate the sympathetic nervous system and the renin–angiotensin system mainly to increase the circulating levels of catecholamines and angiotensin II, respectively. These vasoactive hormones not only elevate blood pressure but also promote the function of subcellular organelles, augment contractile activity, and induce cardiac hypertrophy [5,6,7,37]. Such beneficial effects of these hormones are mediated through the activation of both α- and β-adrenoceptors as well as angiotensin II receptors and involve the activation of various kinases such as protein kinase A, Ca^2+^-calmodulin dependent kinase, protein kinase C and mitogen-activated protein kinase to promote protein synthesis in the myocardium [38,39,40,41]. There also occurs an increase in ventricular diastolic pressure as well as ventricular wall tension, which activate macrophages, fibroblasts, and non-myocyte cells in the myocardial interstitium to release different cytokines and growth factors [42,43,44,45,46,47,48]. In addition, several other neuro-endocrine systems including pituitary, endothelium and platelets are also activated to release vasoactive hormones such as vasopressin, endothelin, and serotonin in the circulation [49,50,51,52]. Thus, different vasoactive hormones at early stages of myocardial infarction can be seen to induce adaptive (physiological) cardiac hypertrophy, stimulate cardiac metabolism and improve cardiac function through their respective receptor mediated signal transduction mechanisms.

**Figure 2:**
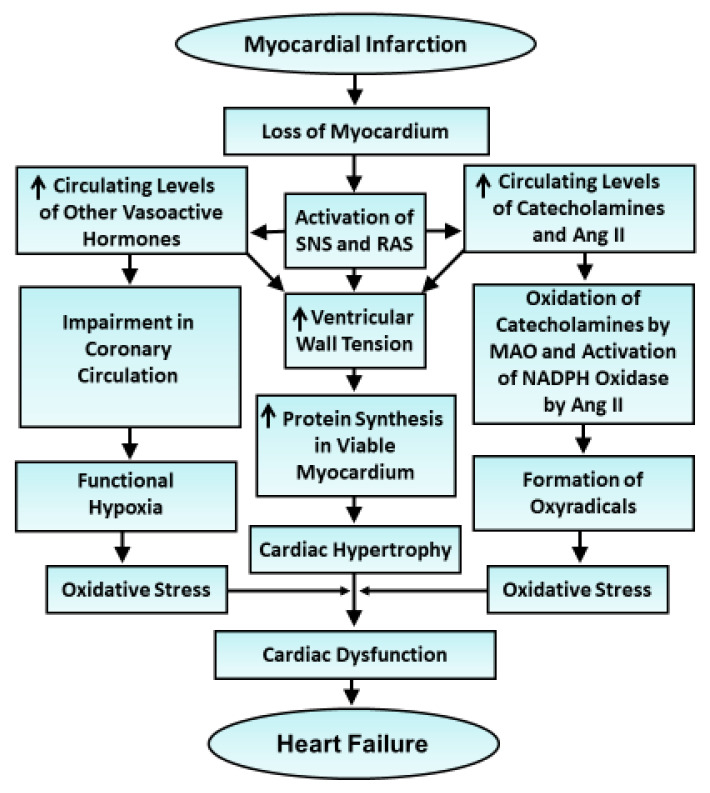
Schematic representation of events for the development of cardiac hypertrophy and oxidative stress as well as the occurrence of cardiac dysfunction in heart failure due to myocardial infarction. SNS—sympathetic nervous system; RAS—renin-angiotensin system; Ang II—angiotensin II; MAO—monoamine oxidase.

Vasoactive hormones generate some amount of oxyradicals in hypertrophied myocardium but the presence of high levels of endogenous antioxidants does not permit the occurrence of oxidative stress [53,54]. However, when the activities of antioxidants become saturated with excessive amounts of oxyradicals or the levels of antioxidants become depressed, there occurs oxidative stress for the development of cardiac dysfunction [55,56,57,58,59]. It should be noted that the increased oxyradical formation may occur due to the activation of NADPH oxidase by angiotensin II and endothelin as well as during the oxidation of catecholamines and serotonin by monoamine oxidase. The combination of oxyradicals with NO, produced by elevated levels of endothelial nitric oxidase in hypertrophied hearts [55], has also been reported to produce nitrosative stress which is known to exert adverse effects on the heart. In addition, defects in mitochondrial electron transport as a consequence of metabolic derangements as well as functional hypoxia, upon prolonged exposure of hypertrophied myocardium to vasoactive hormones, have been shown to contribute to the development of oxidative stress [60,61,62,63,64,65,66]. Since oxidative stress and nitrosative stress have been demonstrated to increase Ca^2+^-influx, activate different proteases and produce alterations in subcellular proteins gene expression directly or indirectly, these pathological entities are considered to induce subcellular remodeling, Ca^2+^-handling abnormalities and cardiac dysfunction in hypertrophied hearts due to myocardial infarction [7,10,67,68,69,70]. Thus, the development of oxidative stress has been suggested to play a critical role in the transition of cardiac hyper-trophy to heart failure due to myocardial infarction. Some events depicting different mechanisms in this regard are shown in Figure 2.

### 2.2. Development of Heart Failure Due to Pressure Overload and Volume Overload

Both pressure overload and volume overload are known to induce cardiac hypertrophy and heart failure upon increasing ventricular pressure as well as ventricular wall tension as a consequence of elevating afterload and preload on the heart, respectively [14,15,25,26,71,72,73,74]. It is now well known that pressure overload occurs in some pathological conditions such as hypertension and aortic or mitral valve stenosis, where the heart develops concentric hypertrophy [71,72,73]. On the other hand, volume overload is seen in some clinical situations including mitral valve or aortic valve regurgitation as well as ventricular septal defect, where the heart develops eccentric hypertrophy [71,72,75,76]. The increase in ventricular wall tension is considered to activate the sympathetic nerve endings, cardiac (local) renin–angiotensin system, endothelium, and several other non-myocytes, present in the myocardial interstitium, to release different vasoactive hormones such as norepinephrine, angiotensin II, and endothelin as well as cytokines and growth factors [31,42,43,44,45,51,77]. Although both the sympathetic nervous system and the peripheral renin–angiotensin system are also activated under situations simulating pressure overload or volume overload [77,78,79,80,81,82,83], exact mechanisms for the release of catecholamines and angiotensin II by these interventions are not clear at present. Nonetheless, both catecholamines and angiotensin II as well as endothelin have been reported to induce cardiac hypertrophy through their respective receptor-mediated signal transduction mechanisms [84,85,86,87]. In this regard, cardiac hypertrophy due to catecholamines is elicited by the activation of both β-adrenoceptor–Gs protein–adenylyl cyclase and α-adrenoceptor–Gq protein–phospholipase C pathways whereas that induced by angiotensin II involves Ang II receptor–Gq protein–phospholipase C system. Furthermore, the involvement of Gq protein–phospholipase C pathway has also been shown to occur due to the activation of endothelin receptors. It is noteworthy that heart function has been reported to be unaltered due to volume overload but is augmented because of pressure overload at early stages of cardiac hypertrophy, indicating differences in the regulatory mechanism participating upon the induction of these pathological stimuli [25].

**Figure 3:**
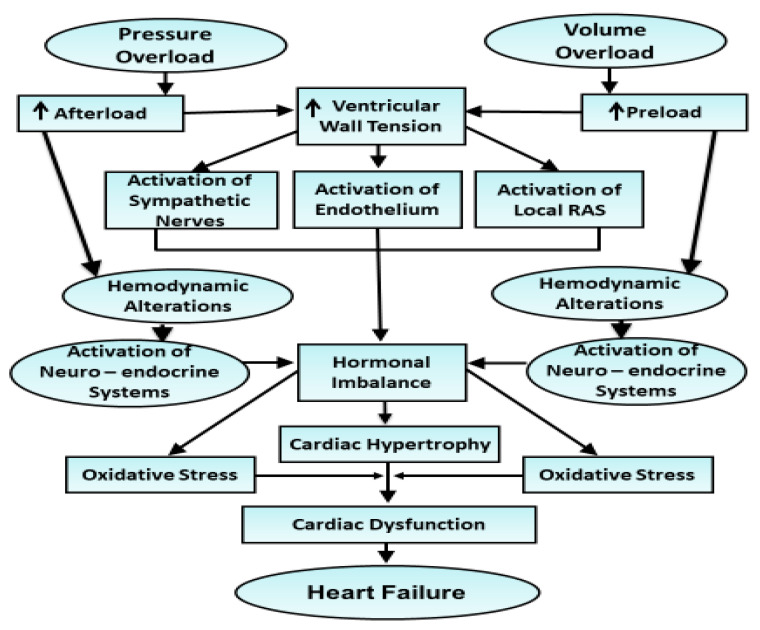
Schematic representation of events for the development of cardiac hypertrophy and oxidative stress as well as the occurrence of cardiac dysfunction in heart failure due to pressure overload or volume overload. RAS—renin–angiotensin system.

Despite differences in cardiac remodeling (concentric versus eccentric cardiac hypertrophy) upon the induction of pressure overload or volume overload, both forms of hemodynamic overload exhibit cardiac dysfunction and heart failure over a prolonged period [71,72,73,74,88,89,90,91]. Several mechanisms including adverse cardiac remodeling, subcellular defects, metabolic derangements, Ca^2+^-handling defects, inflammation, and oxidative stress have been proposed to explain the transition of adaptive (compensated or physiological) hypertrophy to maladaptive (decompensated or pathological) hypertrophy as well as the progression of cardiac hypertrophy to heart failure due to pressure overload or volume overload [15,26,32,48,76]. Furthermore, contractile dysfunction in the failing hearts has been shown to be associated with defects in subcellular organelles for Ca^2+^-handling in cardiomyocytes [7,10,75,82,85,92]. The transition of cardiac hypertrophy to heart failure was also observed due to abnormalities in extracellular matrix proteins as a consequence of the activation of metallomatrix proteases [7,93,94]. Development of apoptosis in cardiomyocytes due to elevated levels of pro-inflammatory cytokines such as TNF-α has been reported to serve as a mechanism of heart failure due to pressure or volume overload [60,95,96,97]. Defects in the β-adrenoceptor signal transduction have also been demonstrated to be associated with the development of heart failure due to these pathological situations [25,98,99,100,101]. In addition, the occurrence of oxidative stress due to elevated levels of vasoactive hormones has been shown to play a major role for the induction of contractile dysfunction in hypertrophied heart [55,56,57,58,59,102,103,104,105]. Thus, it is evident that complex events may be participating in the genesis of cardiac hypertrophy and heart failure due to pressure overload or volume overload. However, a simplified scheme representing these events is shown in Figure 3. 

## 3. Generation of Oxyradicals, Redox Signaling and Consequences of Oxidative Stress in Failing Hearts

An in-depth analysis of the above observations indicates that various changes in subcellular and metabolic mechanisms during the development of cardiac function and heart failure due to myocardial infarction or hemodynamic overload are associated with the generation of oxyradicals. Several other investigators have suggested that different molecular and cellular alterations occur in cardiac hypertrophy and heart failure as a consequence of oxidative stress [106,107,108,109,110,111,112,113]. Since cardiac hypertrophy is an adaptive process; it appears that the formation of a small amount of oxyradicals may not be sufficient for the development of oxidative stress at early stages of pathological stimulus. However, it may generate redox-sensitive signaling to activate the hypertrophic process in the myocardium. On the other hand, high levels of oxyradicals generated due to a prolonged period of pathological stimulus can be seen to result in oxidative stress, adverse cardiac remodeling, cardiac dysfunction, and heart failure. Such a dual role of oxyradical generation is consistent with other pathogenic mechanisms underlying other cardiovascular diseases including cerebral cavernous malformation disease [114]. The small amount of oxyradicals and oxidants, which are formed due to the activation of NADPH oxidase as well as metabolic stimulation and subsequent mitochondrial electron-transport uncoupling as a consequence of the elevated plasma levels of angiotensin II at early stages of pathological stimulus, are removed by different oxyradical scavengers, superoxide dismutase, and catalase, as well as antioxidants [108,110,112]. This condition initiates the redox-sensitive signaling for the activation of hypertrophic process as well as modulation of subcellular activities in cardiomyocytes. A small amount of superoxide anion is also removed by its interaction with NO, which is produced by endothelial nitric oxide synthase at early stages, but this reaction then results in the formation of peroxynitrite at later stages and exert adverse effects [115]. Likewise, low levels of oxyradicals activate nuclear factor erythroid 2-related factor 2 (Nrf2) antioxidant pathway for producing adaptive responses at early period but this master Nrf2 defense pathway has been shown to sensitize cells to oxidative challenges at later stages [114]. Thus, it appears that the adaptive responses of redox-sensitive signaling during the development of cardiac hypertrophy are dependent upon the type, duration, and magnitude of pathological stimulus.

**Figure 4:**
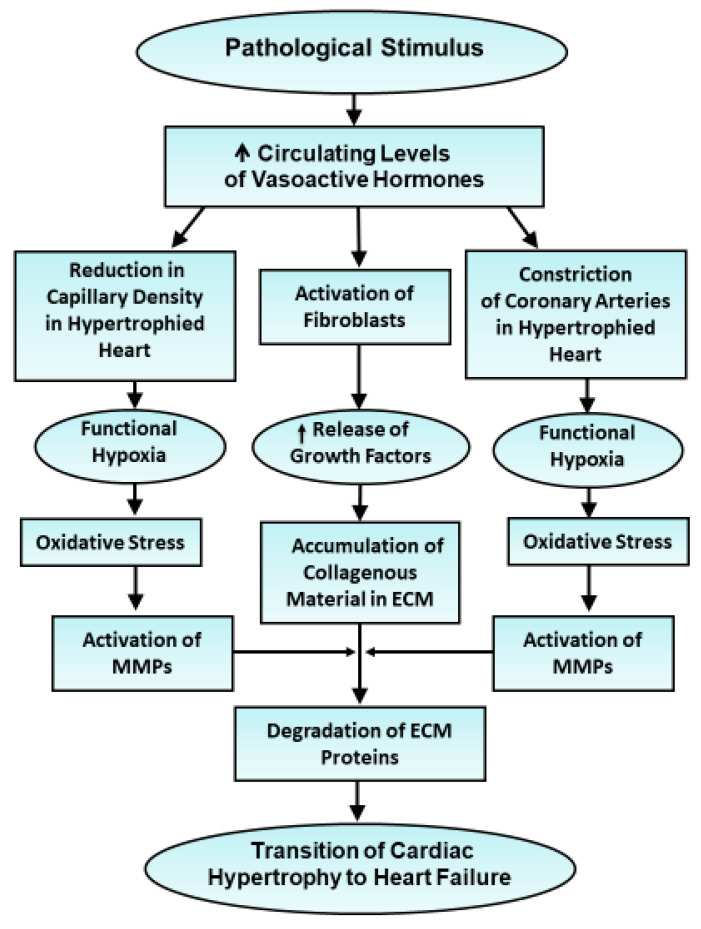
Schematic representation of events depicting the involvement of reduction in capillary density, constriction of coronary arteries and activation of fibroblasts in the hypertrophied myocardium for the transition of cardiac hypertrophy to heart failure due to prolonged elevated levels of circulating hormones. ECM—extracellular matrix; MMPs—metallomatrix proteases.

Both experimental and clinical observations have suggested that oxidative stress in hypertrophied myocardium is increased in heart failure [56,57,58,102,103,104,105,106]. Although the level of antioxidants is also increased in hypertrophied hearts [53,74], it appears that elevated levels of antioxidants at late stages of hypertrophy may not be sufficient to prevent the occurrence of oxidative stress and development of cardiac dysfunction. A large amount of oxyradicals is considered to be formed by a wide variety of mechanisms at late stages of cardiac hypertrophy. In this regard, it is noteworthy that elevated levels of both angiotensin II and endothelins have been shown to activate NADPH oxidase [63,64] whereas catecholamines are oxidized by monoamine oxidase [61,62] to produce oxyradicals in the myocardium. The expression of both NADPH oxidase and monoamine oxidase has been shown to be increased in failing hearts [55]. The vasoactive hormones are also known to impair blood flow to the heart and induce functional hypoxia due to constriction of the coronary arteries as well as reduction in capillary density in hypertrophied hearts [42,43,44,45]. The hypoperfusion thus produced can be seen to increase the production of oxyradicals upon inducing defects in the mitochondrial electron transport due to hypoxic insult and contribute to the development of oxidative stress [37,38,39,40,41,42,43,44,45,46,47,48,49,50,51,52,53,54,55]. Furthermore, elevated levels of vasoactive hormones have also been reported to activate fibroblasts in the cardiac interstitium to release different growth factors and metallomatrix proteases [31,43,44]. While the growth factors promote the accumulation of collagenous proteins in the extracellular matrix for providing support to hypertrophied hearts [37,42,46], the activation of metallomatrix proteases by oxidative stress results in degradation of the glycocalyx proteins and subsequent cardiac dysfunction [37,44,60]. Accordingly, oxidative stress upon activating metallomatrix proteases has been suggested to play a critical role in the transition of stable cardiac hypertrophy to heart failure [32,60,93]. Some of the events involved in this process are depicted in Figure 4. 

**Figure 5:**
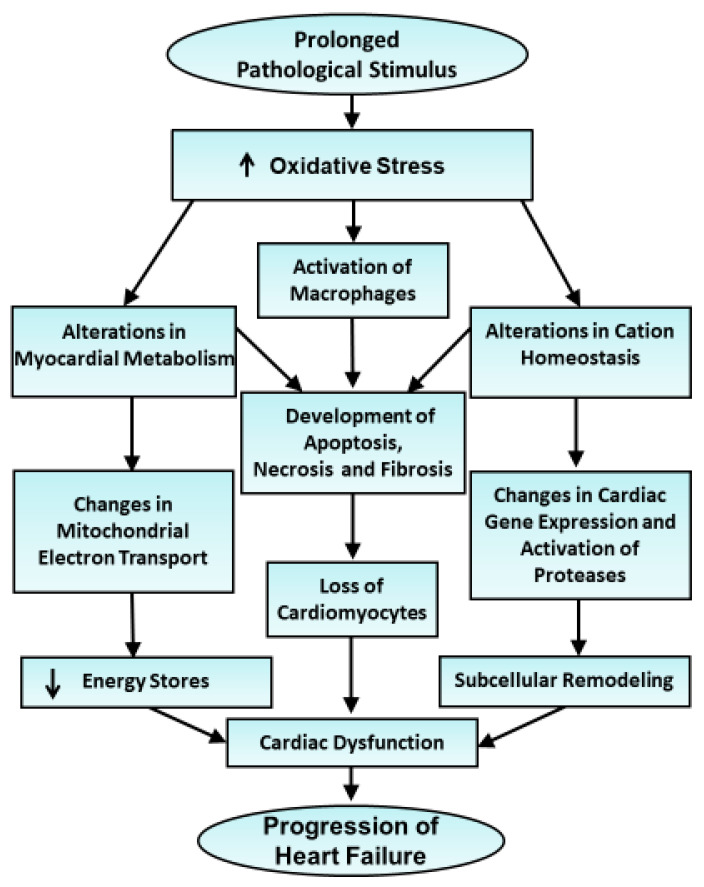
Schematic representation of events depicting the involvement of alterations in myocardial metabolism, cation homeostasis as well as activation of macrophages for the occurrence of cardiac dysfunction and progression of heart failure due to the development of oxidative stress hormones in hypertrophied heart for a prolonged period.

The participation of oxidative stress in the genesis of cardiac dysfunction is attributed to the development of several cardiac abnormalities in hypertrophied heart. Although adverse cardiac remodeling is generally considered to explain the occurrence of heart failure [5], it has been argued that a wide variety of changes in subcellular organelles including sarcolemma, sarcoplasmic reticulum, mitochondria, and myofibrils may be more intimately related to the development of contractile dysfunction during the progression of heart failure [7,10,116,117]. Such subcellular defects during the development of heart failure have been shown to occur because of alterations in cation homeostasis, increased concentration of intracellular Ca^2+^, activation of proteases, and changes in cardiac gene expressions [7,70,118]. Particularly, defects in sarcolemma and sarcoplasmic reticulum may results in Ca^2+^-handling abnormalities in myocytes whereas those in myofibrils and mitochondria are associated with changes in contractile properties and energy production in the failing hearts, respectively. It should be mentioned that increased concentration of Ca^2+^ in the failing heart is known to result in mitochondrial Ca^2+^-overload and impair the generation of ATP. In fact, oxidative stress has also been associated with marked alterations in myocardial metabolism and mitochondrial electron transport system for depression in energy stores in the failing heart [37,55,60,65,66]. In addition, oxidative stress has been demonstrated to induce loss of cardiomyocytes in the heart by inducing apoptosis, necrosis, and fibrosis as a consequence of myocardial inflammation due to activation of macrophages in the cardiac interstitium and release of different cytokines [31,37,48,55,97,119,120,121,122]. Accordingly, oxidative stress generated in hypertrophied heart can be seen to induce cardiac dysfunction through a complex set of mechanisms and may result in the progression of heart failure. A schematic representation of these events is shown in Figure 5. In view of the critical role of oxidative stress in the pathophysiology of cardiac dysfunction in heart failure, different antioxidants have been suggested to exert beneficial effects for the treatment of this devastating disease [54,55,56,57,58]. Although some clinical studies have been supportive of this concept, other clinical trials with antioxidants have failed to show any conclusive benefit of these interventions for the treatment of heart failure [123,124]. Several investigators have discussed in detail the inability of different non-specific antioxidants to exert beneficial effects in heart failure [107,108,109,115]. Thus, better-targeted and more effective antioxidants need to be developed for improved therapy of this disease. Since, oxidative stress in heart failure is also accompanied by nitrosative stress [115] and inflammation [120,121], it is likely that a combination therapy with antioxidants may prove more appropriate.

## 4. Evidence for the Implications of Oxidative Stress in Cardiac Dysfunction and Subcellular Remodeling

In view of the association of cardiac dysfunction and oxidative stress during the development of heart failure, various investigators have emphasized the role of oxidative stress in the genesis of subcellular and metabolic defects for the occurrence of contractile abnormalities in hypertrophied hearts [6,10,37,55,104,106]. However, it is not clear whether the occurrence of cardiac dysfunction is a consequence of events associated with cardiac hypertrophy or is due to some direct action of oxidative stress on cardiomyocytes per se. Further discussion in this report is thus focused to provide evidence that the generation of oxidative stress in non-hypertrophied heart leads to the development of subcellular alterations and cardiac dysfunction [125,126,127,128,129]. Different oxygen reactive species, which result in the development of oxidative stress, have been reported to be involved not only in inducing changes in cardiac contractile activity but are also considered to be mediators of the myocardial cell injury [130,131,132,133,134]. This article will examine the effects on oxidative stress generation on changes in subcellular activities and cardiac function in ischemic reperfused hearts in the absence and presence of different oxyradical scavengers and antioxidant interventions. The existing literature for changes in cardiac function and subcellular activities will also be analyzed upon perfusing the hearts with some oxidative stress generating systems. Furthermore, different oxyradicals will be shown to exert direct actions on the activities of cardiac subcellular organelles. Such a detailed examination of the effects of oxidative generating systems on cardiac contractile activities, subcellular remodeling and Ca^2+^-handling in cardiomyocytes will further support the role of oxidative stress in the development and progression of heart failure.

### 4.1. Alterations in Cardiac Function and Subcellular Activities in Ischemic Reperfused Hearts

Since ischemia-reperfusion is well known to generate oxyradicals [126,135], some studies have examined the relationship between changes in cardiac function and subcellular alterations upon subjecting the heart to ischemia-reperfusion in the absence and presence of different oxyradical scavengers or antioxidants. Various parameters such as left ventricular systolic pressure, rate of contraction, and rate of relaxation were markedly depressed whereas left ventricular end diastolic pressure was increased in the ischemic reperfused hearts [136,137,138,139]. These alterations in cardiac function were associated with marked depressions in the sarcolemmal Na^+^-K^+^ ATPase activity [136,140], Ca^2+^-pump activity, and Na^+^-Ca^2+^ exchange activity [141], as well as β-adrenoceptor–adenylyl cyclase mediated pathway [142]. Dramatic reduction in the sarcoplasmic reticulum Ca^2+^-uptake, Ca^2+^-pump ATPase, and Ca^2+^-release [137] as well as Ca^2+^/calmodulin protein kinase activities [143] were observed in the ischemic reperfused hearts. Furthermore, depressed cardiac function was seen to be associated with marked alterations in mitochondrial oxidative phosphorylation [138] as well as myofibrillar ATPase activities [139]. All these changes in cardiac function as well as sarcolemma, sarcoplasmic reticulum, mitochondria, and myofibrils due to ischemia-reperfusion were attenuated by the presence of an oxyradicals scavenging mixture (superoxide dismutase plus catalase) in the perfusion medium [136,137,138,139,140,141,142,143]. Furthermore, treatments of the hearts with antioxidants such as N-acetylcysteine (NAC) and N-mercaptopropionylglycine (MPG) were also found to partially or fully prevent the ischemia-reperfusion induced alterations in cardiac function as well as different subcellular organelles [138,139,144]. 

Hearts subjected to ischemia-reperfusion were observed to exhibit varying degrees of depressions in mRNA levels for sarcoplasmic reticular Ca^2+^-pump ATPase and Ca^2+^-release channels [137], sarcolemmal Na^+^-K^+^ ATPase α_2_, α_3_, and β_1_ isoforms [140], and myofibrillar myosin heavy chain α- and β-isoforms as well as myosin light chain 1 [139]. All theses changes in gene expression for subcellular proteins due to ischemia-reperfusion were attenuated by superoxide dismutase plus catalase indicating the involvement of oxidative stress for subcellular remodeling [137,139,140]. The ischemia-reperfusion induced alterations in cardiac function, subcellular activities, and subcellular gene expressions are also attenuated by ischemic preconditioning [145,146,147,148], which is known to depress the development of oxidative stress in the myocardium [149,150]. Furthermore, the observed depressions in sarcolemmal and sarcoplasmic reticular enzyme activities in the ischemic reperfused hearts have been reported to be due to the activation of Ca^2+^-dependent protease, calpain [151,152,153,154]. Since oxidative stress due to ischemia-reperfusion has been demonstrated to produce intracellular Ca^2+^-overload and activate different proteolytic enzymes [55,155], it is likely that changes in subcellular activities are a consequence of an indirect effect of oxidative stress. 

### 4.2. Alterations in Cardiac Function and Subcellular Activities Due to Oxyradical Generating System or H_2_O_2_

The effects of oxidative stress on cardiac function and subcellular activities have been examined by perfusing the heart with either xanthine plus xanthine oxidase (a well known oxyradical generating system) or H_2_O_2,_ an oxidant [136,137,138,139,140,141,144,156,157,158,159]. The depression in cardiac function upon perfusion with xanthine plus xanthine oxidase or H_2_O_2_ was associated with decreased activities of sarcolemmal Na^+^-K^+^ ATPase, Na^+^-Ca^2+^ exchange, and Ca^2+^-pump ATPase as well as β-adrenoceptor–adenylyl cyclase system [136,140,141,156,157,158,159]. Likewise, sarcoplasmic reticular Ca^2+^-uptake and release activities, myofibrillar ATPase and mitochondrial oxidative phosphorylation activities were also reduced upon perfusing the hearts with H_2_O_2_ or xanthine plus xanthine oxidase [137,138,139]. These alterations in sarcolemma, sarcoplasmic reticulum, myofibrils, and mitochondria due to oxyradicals and oxidants were attenuated by the presence of superoxide dismutase plus catalase in the perfusion medium [136,137,138,141,156,157,158,159]. Furthermore, treatments of hearts with antioxidants, NAC and MPG, were observed to attenuate xanthine plus xanthine oxidase induce depressions in cardiac function as well as sarcoplasmic reticular Ca^2+^-uptake and Ca^2+^-release activities [144]. These observations provide evidence that oxidative stress is intimately involved in inducing cardiac dysfunction and subcellular defects. 

### 4.3. Effects of Oxyradical Generating System and H_2_O_2_ on Subcellular Activities 

To examine whether oxidative stress induces subcellular alterations directly, various isolated organelles were incubated with systems known to generate different species of reactive oxygen. Both H_2_O_2_ and oxyradical generating systems were found to depress sarcolemmal Na^+^-K^+^ ATPase, Na^+^-Ca^2+^ exchange, and Ca^2+^-pump activities by promoting lipid peroxidation and modifying the sulfhydryl groups [160,161,162,163]. Likewise, Ca^2+^-uptake and Ca^2+^-pump ATPase activities in the sarcoplasmic reticulum were decreased by superoxide and hydroxyl radicals as well as H_2_O_2_ [164,165,166,167]. It is noteworthy that different reactive oxygen species were observed to depress the sarcolemmal Ca^2+^-channel binding activity; the effect by superoxide radical was prevented by superoxide dismutase whereas that by H_2_O_2_ and hydroxyl radicals was prevented by catalase and mannitol, respectively [168]. The effects of oxyradicals and H_2_O_2_ on sarcolemmal Ca^2+^-ecto ATPase, ATP-independent Ca^2+^-binding, β-adrenergic density, and adenylyl cyclase were of biphasic nature and oxyradical species specific [169,170,171]. Various oxyradical generating systems were observed to impair mitochondrial oxidative phosphorylation and reduce myofibrillar ATPase activity [138,172]. These observations are consistent with the view that various oxyradicals and oxidants modify the activities of different subcellular organelles directly in the heart. In addition, as described above, oxidative stress may also alter subcellular activities indirectly by affecting cardiac gene expression as well as activating different proteolytic enzymes as a consequence of increased concentration of intracellular Ca^2+^ in cardiomyocytes. 

## 5. Conclusions

**Figure 6:**
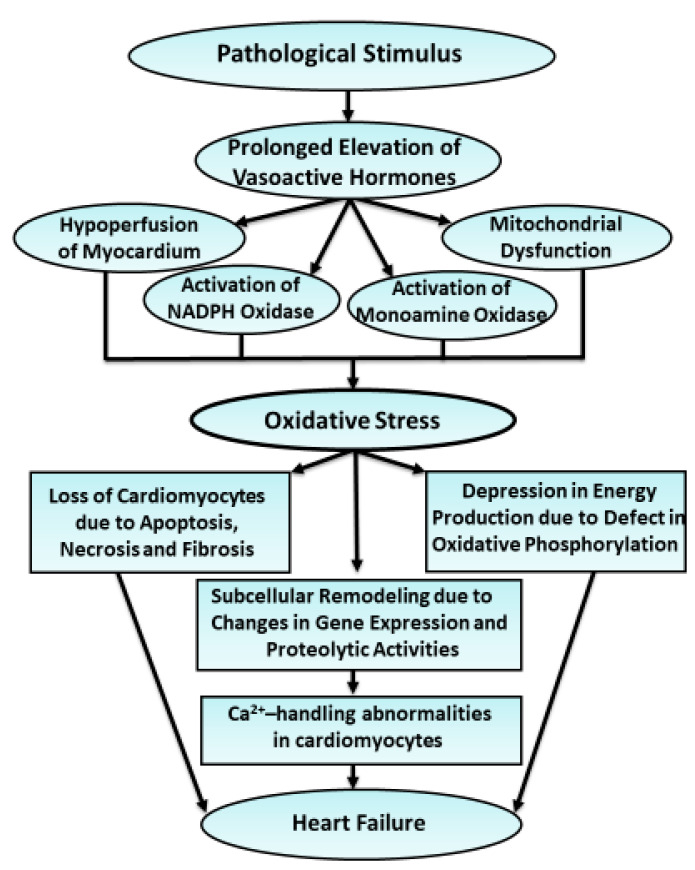
Schematic representation of events depicting the generation of oxidative stress due to prolonged exposure of hypertrophied heart to vasoactive hormones as well as the occurrence of cardiac abnormalities due to oxidative stress for the development of heart failure in hypertrophied myocardium.

It is evident that heart failure due to myocardial infarction, pressure overload, or volume overload is mainly associated with elevated levels of plasma catecholamines, oangiotensin II and endothelin. These vasoactive hormones stimulate their receptor-mediated signal transduction pathways and induce cardiac hypertrophy, which is a beneficial mechanism for maintaining or augmenting heart function at initial stages. A small amount of oxyradicals is also generated during early periods of hypertrophic process; these radicals are readily removed by the endogenous scavengers for maintaining redox homeostasis. However, prolonged exposure of hypertrophied heart to pathological stimuli and subsequent high levels of circulating hormones has been demonstrated to promote the development of oxidative stress as a consequence of functional hypoxia due to constriction of the coronary arteries, reduction in the capillary density, myocyte inflammation, metabolic derangements, and mitochondrial dysfunction. Furthermore, activation of NADPH oxidase by hormones such as angiotensin II and endothelins, and oxidation of catecholamines by monoamine oxidase also participate in the generation of oxidative stress. Oxidative stress has been suggested to cause Ca^2+^-handling abnormalities in association with subcellular remodeling, defect in energy production, inflammation, apoptosis, fibrosis, and loss of cardiomyocytes; these abnormalities are considered to result in cardiac dysfunction and heart failure. Such events showing adverse cardiovascular effects of diverse pathological stimuli for the generation of oxidative stress and subsequent myocardial abnormalities are shown in Figure 6. Evidence has also shown that depression in cardiac function and associated subcellular defects upon exposure of the heart to some oxidative stress generating systems were attenuated by oxyradical scavengers and antioxidants. These observations support the view that the development of oxidative stress in hypertrophied heart is an important mechanism for transition of cardiac hypertrophy to heart failure. Furthermore, the details regarding the molecular and cellular effects as well as Ca^2+^-handling abnormalities due to oxidative stress provide compelling evidence for the potential use of antioxidants for the treatment of heart failure. Thus, various antioxidants or interventions for increasing the antioxidant reserve in hypertrophied myocardium may produce beneficial effects in preventing the occurrence of cardiac dysfunction and delaying the progression of heart failure. However, the results from some clinical trials of antioxidants in heart failure patients have been disappointing, perhaps due to non-specific nature of these agents. Thus, there is real challenge for the cardiovascular community to develop target-orientated specific antioxidants for improved therapy of heart failure.

## Data Availability

Data is contained within the article.
